# ASPIRE to new horizons

**DOI:** 10.1093/immhor/vlag009

**Published:** 2026-04-22

**Authors:** Bonnie N Dittel

**Affiliations:** Versiti Blood Research Institute, P.O. Box 2178, Milwaukee, WI 53201-2178, United States

As a scientist in training, I often wondered what it truly meant to be recognized as an immunologist. Beyond publishing rigorous and insightful research, how does one gain visibility and acknowledgment within a field that is rapidly expanding? The American Association of Immunologists (AAI) has taken a meaningful step toward answering that question. It recently introduced a new award category designed to spotlight early career investigators within the first 5 years of establishing their laboratories. Fittingly named the ASPIRE Award, it gives recipients the opportunity to present their research in a dedicated session at the AAI annual meeting.

**Figure:**
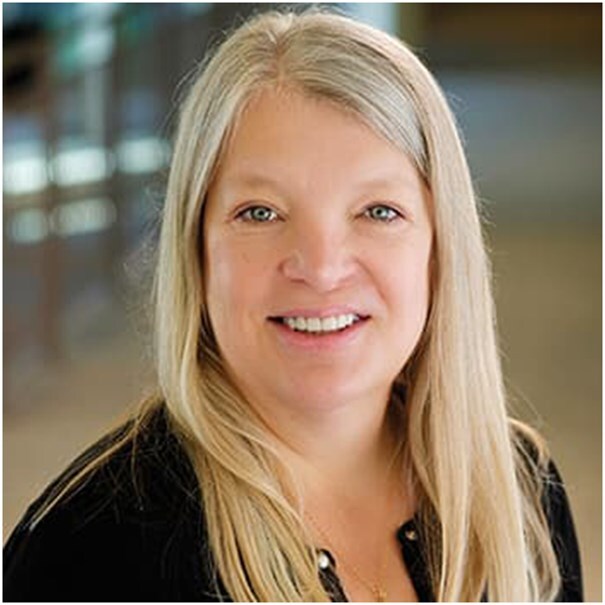
Dr. Bonnie Dittel, PhD, Editor-in-chief *ImmunoHorizons*; Senior Investigator at the Versiti Blood Research Institute in Wisconsin; and Professor of Microbiology and Immunology at the Medical College of Wisconsin.

*ImmunoHorizons* is proud to help elevate these rising leaders and introduce their contributions to the broader immunology community. The ASPIRE winners hail from the West Coast to the East Coast and everywhere in between, showing that excellent immunology is being pursued across all of North America. Each ASPIRE Award recipient was invited to write a comprehensive review capturing the evolution and impact of their research, accompanied by a brief biographical sketch. This special issue highlights 5 of these exceptional scientists, spanning topics from nasal humoral immunity and tissue-resident responses to neuroimmune circuitry and big data–driven discovery—areas that represent some of the most exciting and rapidly developing frontiers in immunology today.

The first article in this collection comes from the laboratory of Jenna Guthmiller at the University of Colorado Anschutz Medical Campus and brings our focus to the front lines of immune defense—the nasal mucosa.^1^ This review provides a comprehensive overview of the structure and immunological architecture of the nasal cavity and its associated lymphoid tissues, where local humoral responses serve as critical protection against respiratory pathogens, particularly influenza and SARS-CoV-2. Given its dominant role in this compartment, the authors offer an insightful discussion of secretory IgA biology before turning to one of the most timely and exciting areas in the field: strategies for designing effective intranasal vaccines. For readers eager to explore influenza further, we also direct your attention to the *ImmunoHorizons* Thanksgiving Special Issue on avian influenza (November 2025).

The second review in this series, from the laboratory of Pamela Rosato at the Geisel School of Medicine at Dartmouth College, shifts our focus to T cell–mediated immunity with a panoramic look at tissue-resident memory CD8^+^ T cells (T_RM_) in the brain.^2^ Despite the once-popular belief that the central nervous system is immune privileged, T_RM_ cells can enter the central nervous system and are essential for keeping resident viruses in check. This review navigates the complex immune architecture of the brain, clarifying where T_RM_ cells reside and how they protect, while also confronting their more troublesome side in neuroinflammation and neurodegeneration. The article concludes with a discussion of T_RM_ heterogeneity, proof that even brain-resident T cells are not all of one mind. A highly engaging read, and one that won’t leave you with a headache, neurologic or otherwise.

Anna-Maria Globig, of the Allen Institute for Immunology, also dives into the intricate relationship between the immune and nervous systems, this time with a spotlight on the peripheral nervous system.^3^ Although these 2 networks are woven through every organ of the body, remarkably little is understood about how they communicate, collaborate, or occasionally clash. This review maps neuroimmune interactions across a broad landscape of challenges, including cancer, viral and bacterial infections, parasitic disease, and allergies, even those uninvited dust mites currently residing in your mattress. The discussion then turns to immune-mediated diseases such as psoriasis and inflammatory bowel disease, culminating in thoughtful clinical and translational perspectives. In keeping with the *ImmunoHorizons* theme of “On the Horizon,” the review closes with a forward-looking view of where the field is headed. So, the next time your nerves kick in, consider that your immune system just might be in the conversation as well.

Brandon DeKosky, of The Ragon Institute of Mass General, MIT, and Harvard, takes on the expansive world of “big data,” demonstrating how high-throughput display technologies, next-generation sequencing, and machine learning are transforming our ability to characterize immune receptor repertoires and their functional properties.^4^ This review spans both T-cell receptors and antibodies, offering historical context alongside a contemporary exploration of the unique challenges posed by receptors composed of not one, but 2 chains—proof that immunology has always been a combinatorial sport. The latest advances in the field are clearly presented, outlining both the obstacles and exciting opportunities that come with applying large-scale data analytics to immunology. For readers new to this rapidly evolving area, the review is especially valuable as complex systems are distilled into digestible, comprehensible pieces, no aspirin required.

The final article in the series, from Miguel Reina-Campos at the La Jolla Institute for Immunology, brings us back into the tissue microenvironment with a compelling overview of how high-resolution spatial data can illuminate immune behavior where it matters most at the right place and the right time.^5^ The review offers a clear and accessible introduction to spatial technologies and how they are reshaping our understanding of the immune system in situ. From transcriptomic and metabolomic mapping to proteomic and epigenetic profiling, the potential insights are vast, spanning infectious disease, cellular communication, and beyond. The authors also highlight current knowledge gaps and the innovations poised to bridge them. In keeping with our “On the Horizon” theme, the review concludes with a forward-looking perspective on this rapidly emerging technology still early in its development yet already transforming how we visualize immunity.

Collectively, the work showcased in this special issue demonstrates the breadth, depth, and creativity driving the next generation of immunology. From mucosal defenses at the gateway of infection, to neuroimmune interactions in both the central and peripheral nervous systems, to the power of big data and spatial technologies to reveal the immune system in unprecedented detail, these reviews capture how rapidly our field is evolving. The ASPIRE Award was created to recognize early career investigators who are not only pushing boundaries but redefining them. We are confident that the insights shared here reflect discoveries still in their early chapters—advances that will shape the questions we ask, the tools we use, and the therapies we envision. As these rising leaders continue to explore what lies just beyond the horizon, the future of immunology has rarely looked more promising.
